# Supplementary material for: Genomics-informed isolation and characterization of a symbiotic Nanoarchaeota system from a terrestrial geothermal environment
Source: Nat Commun. 2016 Jul 5;7:12115. doi: 10.1038/ncomms12115 (PMC4935971; doi:10.1038/ncomms12115)
Supplement: Supplementary Information — Supplementary Figures 1-5, Supplementary Table 1 and Supplementary References [file ncomms12115-s1.pdf]

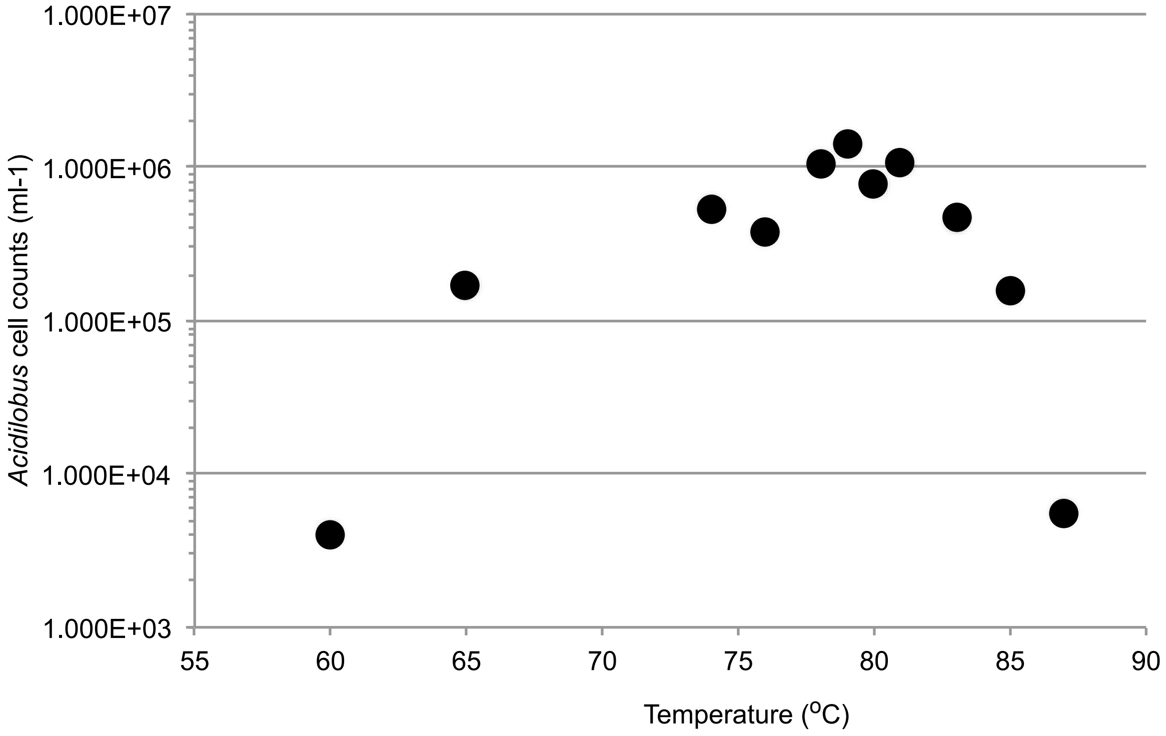


**Supplementary Figure 1.** Effect of temperature on *Acidilobus* sp.7A growth in co-culture with *N. acidilobi,* based on cell density measured after 3 days.

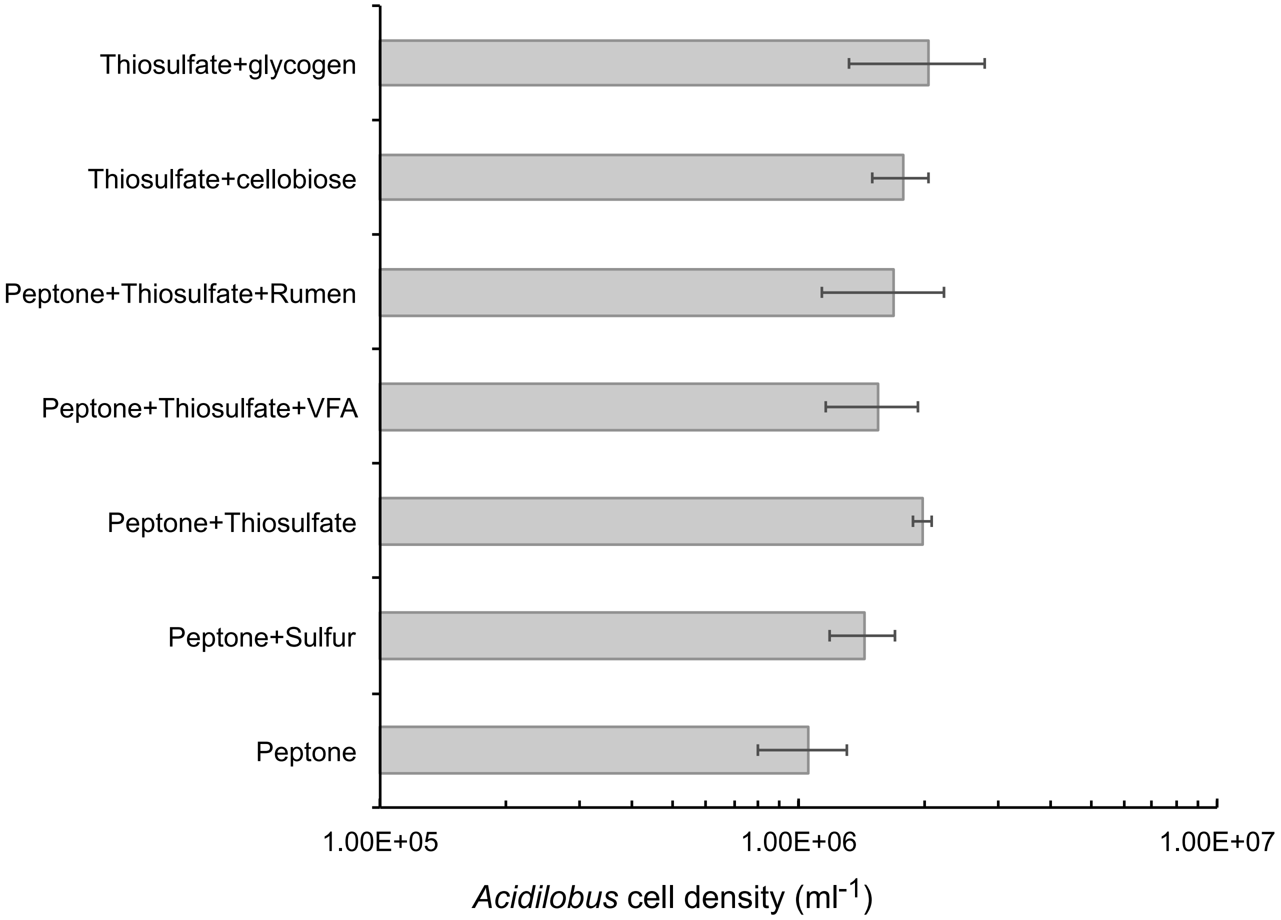


**Supplementary Figure 2.** Effect of variations in media composition on *Acidilobus* growth in co-culture with *N. acidilobi*, based on cell density measured after 3 days. Scale bars correspond to standard deviation based on 3 replicate cultures.

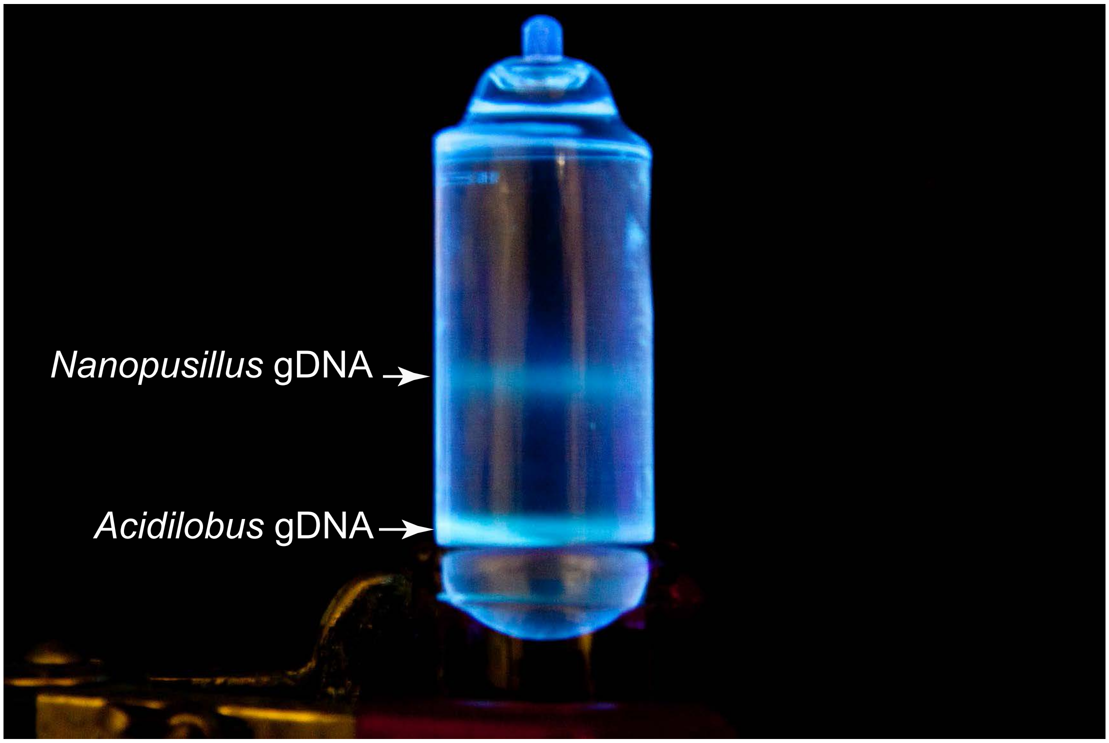


**Supplementary Figure 3**. Separation of *Acidilobus* and *Nanopusillus* gDNA based on equilibrium density ultracentrifugation in bisbenzimide-CsCl gradient

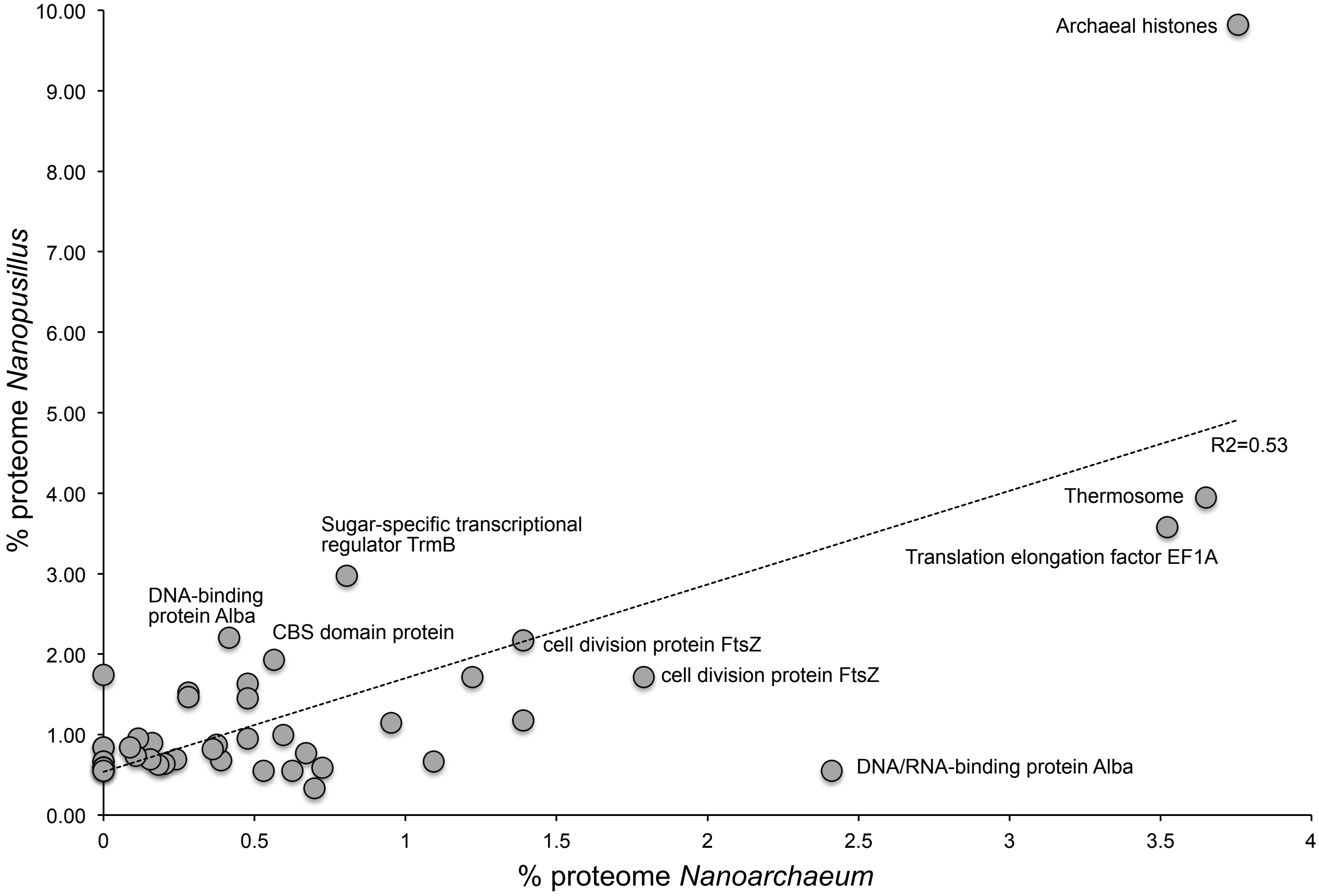


**Supplementary Figure 4**. Comparison of the top 50 relative most abundant proteins in *Nanopusillus acidilobi* versus *Nanoarchaeum equitans* (data from Giannone et al, 2011). The proteins indicated as 0% on the *Nanoarchaeum* axis are not encoded in the genome.

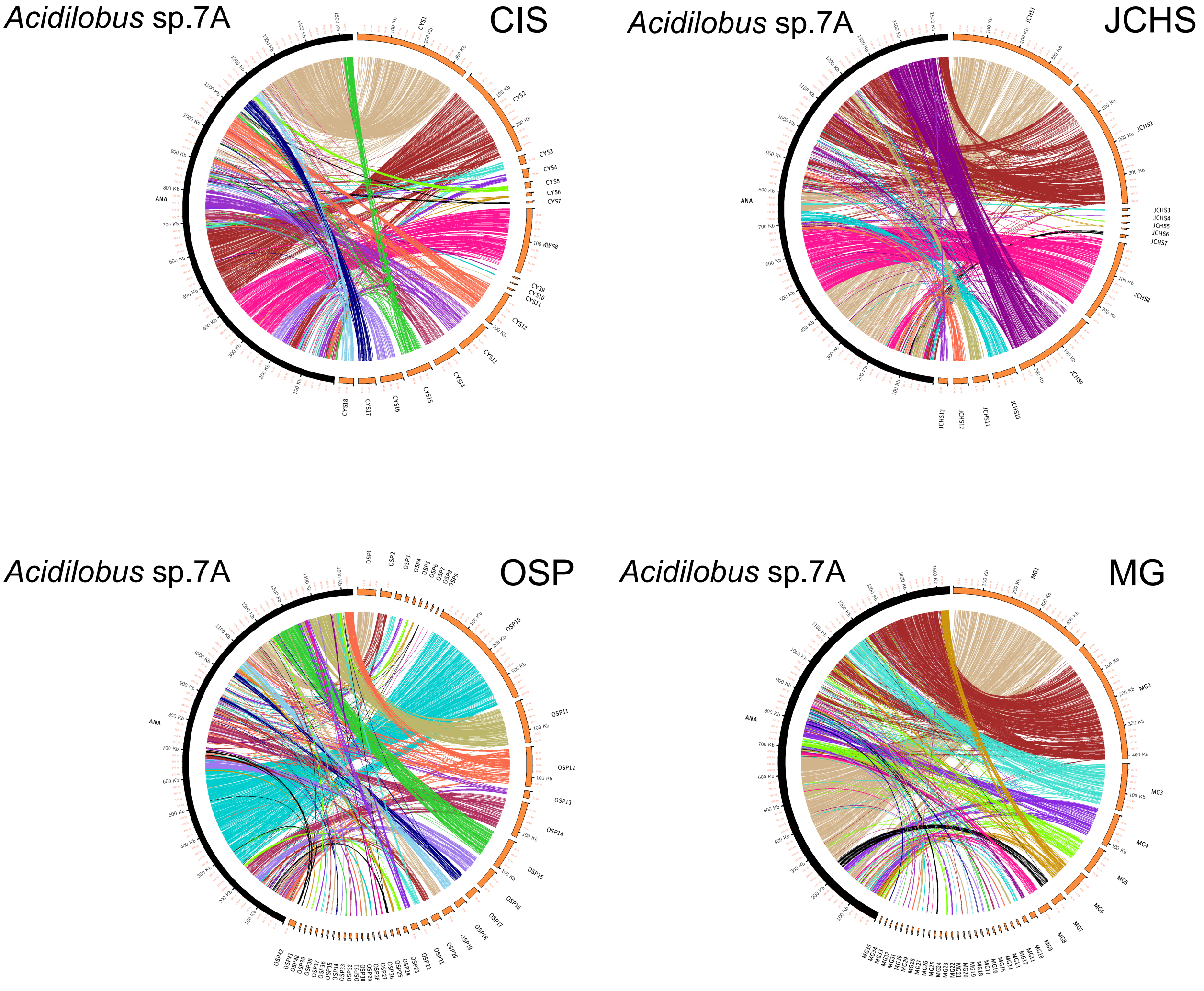


**Supplementary Figure 5**. Circos-based genome alignments between *Acidilobus* sp.7A and *Acidilobus*-like populations from Yellowstone (Jay et al 2014)

| | **N. equitans** | **Nst1** | **N. acidilobi** | **Note** |
|---|---|---|---|---|
| **Size** | 491kb | 593kb (7 contigs) | 606 kb | |
| **G+C %** | 31.6% | 24.3% | 24.3% | |
| **Split Proteins** | | | | |
| Reverse gyrase | NEQ 318-434 | Nst 337-402 | Nps 2030-2705 | Same site |
| Glu-tRNA$^{Gln}$ amidotransferase | NEQ 245-396 | Nst 197-449 | Nps 2280-3415 | Same site |
| Predicted RNA-binding protein | NEQ 438-506 | Nst 176-251 | Nps 30-3140 | Same site |
| Archaeosine tRNA-guanine transglycosylase | NEQ 124-305 | Nst 096-232 | Nps 1135-3235 | Same site |
| RNA polymerase subunit B | NEQ 156-173 | Nst 632-633 | Nps 545-550 | Same site |
| Large helicase-related protein | NEQ 003-409 | Nst 172-239 | Nps 745-3195 | Different site |
| DNA polymerase I | NEQ 068-528 | Nst 417 | Nps 2105 | Not split in *Nst1-Nac* |
| Topoisomerase I | NEQ 045-324 | Nst 174 | Nps 45 | Not split in *Nst1-Nac* |
| P-loop ATPase-acetyltransferase fusion protein | NEQ 096-495 | Nst 401 | Nps 2025 | Not split in *Nst1-Nac* |
| Alanyl-tRNA synthetase | NEQ 211-547 | Nst 054 | Nps 290 | Not split in *Nst1-Nac* |
| Diphthamide synthase sub. DPH2 | - | Nst 222-440 | Nps 3285-2235 | Absent in *N.eq.* |
| Uncharacterized conserved protein (arCOG04253) | - | Nst 474-480 | Nps 2410 | Absent in *N.eq.* |
| **tRNAs** | | | | |
| *cis*-spliced tRNAs | Ile, Met, Trp, Tyr | Ile, Tyr | Ile, Tyr | |
| *trans*-joined tRNAs | iMet, His, Lys, Gln, Glu (2) | none | none | |
| RNase P | Absent | Present | Present | |
| Gluconeogenesis-Glycolysis | Absent | Present | Present | |
| Polyamine biosynthesis | Absent | Present | Present | |
| ATP synthase | Incomplete | Absent | Absent | |
| Glutamate dehydrogenase | Present | Absent | Absent | |

Supplementary Table 1. Comparison of genomic features (split genes and metabolic features) among sequenced Nanoarchaeota.

**Supplementary References**

Giannone, R. J. et al. Life on the edge: functional genomic response of Ignicoccus hospitalis to the presence of Nanoarchaeum equitans. ISME J 9, 101-114 (2015).

Jay, Z. J. et al. Predominant Acidilobus-Like Populations from Geothermal Environments in Yellowstone National Park Exhibit Similar Metabolic Potential in Different Hypoxic Microbial Communities. Appl. Env. Microbiol. 80, 294-305 (2014).
